# Effect of N-Acetylcysteine on Dyslipidemia and Carbohydrate Metabolism in STZ-Induced Diabetic Rats

**DOI:** 10.1155/2018/6428630

**Published:** 2018-01-28

**Authors:** Anderson Kiyoshi Kaga, Pedro Octavio Barbanera, Nágilla Orleanne Lima do Carmo, Lucas Rodolfo de Oliveira Rosa, Ana Angélica Henrique Fernandes

**Affiliations:** Department of Chemistry and Biochemistry, Institute of Biosciences of Botucatu, Sao Paulo State University (UNESP), Rua Professor Doutor Antônio Celso Wagner Zanin s/n, Distrito de Rubião Júnior, 18618-689 Botucatu, SP, Brazil

## Abstract

**Background:**

Type 1 diabetes mellitus (T1DM) is characterized by insulin-deficient production leading to hyperglycemia, which is associated with diabetic complications such as cardiovascular diseases. Antioxidants have been proving a good alternative to diabetic complications, with N-acetylcysteine (NAC) having antioxidant characteristics. The aim of this study was to assess the effect of NAC on the lipid profile and the atherogenic index (AI) in streptozotocin- (STZ-) induced diabetic rats.

**Method:**

32 male Wistar rats (60 days of age) weighting ±250 g were randomly distributed into four groups (*n* = 8): CTRL: control rats; CTRL+NAC: control rats treated with NAC; DM: diabetic rats; DM+NAC: diabetic rats treated with NAC. T1DM was induced using STZ (60 mg/kg, ip; single dose), and NAC (25 mg/kg/day) was administrated by gavage, for 37 days. The animals received chow and water* ad libitum*. After the experimental period, blood and cardiac tissue samples were collected to analyze energetic metabolism, lipid profile, and AI.

**Results:**

NAC decreased (*p* < 0.01) glycemia, energy intake, carbohydrate, and protein consumption in diabetic rats (DM+NAC), when compared with DM, while the alimentary efficiency was improved (*p* < 0.01) in treated diabetic rats (DM+NAC). Diabetic rats treated with NAC decreased (*p* < 0.01) lipid profile and AI in diabetic rats (DM+NAC) when compared to DM.

**Conclusion:**

NAC improves lipid profile and decreases AI in STZ-induced diabetic rats.

## 1. Introduction

In 2015, the International Diabetes Federation [1] estimated a global diabetic population of 415 million, with expectation to reach 642 million by 2040. Approximately 10% of the diabetic population suffer from type 1 diabetes mellitus [2, 3], and it is increasing by 3% every year [4, 5].

Type 1 diabetes mellitus (T1DM) is a pathology mediated by an autoimmune response that destroys the pancreatic *β* cells responsible for insulin production, resulting in a deficiency of this hormone. Because insulin promotes glucose uptake in the cellular environment, individuals with T1DM suffer from hyperglycemia [6].

Glucose is the main oxidizable substrate in various cell types. In a diabetic state, catabolic pathways such as gluconeogenesis and glycogenolysis are activated in an attempt to maintain homeostasis [7]. In this scenario, lipolysis is also increased, which can trigger dyslipidemia, a risk factor for the development of atherosclerosis that affects 97% of diabetic patients [8–10].

Dyslipidemia is characterized by elevated levels of triacylglycerol plasma and total cholesterol, increasing the concentration of very low-density lipoproteins (VLDL-cholesterol) and low-density lipoproteins (LDL-cholesterol) and decreasing the concentration of high-density lipoproteins (HDL-cholesterol) [10, 11].

Studies have shown that an increase in LDL-cholesterol is directly related to the development of atherosclerosis, the inflammatory process triggered by the oxidation of LDL-cholesterol that occurs due to the release of oxidizing agents during the metabolic pathways (advanced glycation end products, protein kinase C pathway, and hexosamine pathway) activated by hyperglycemia [12, 13].

Oxidized LDLs are phagocytosed by macrophages in the subendothelial layer, thereby transforming into foam cells that contribute to the formation of atherosclerotic plaque and, consequently, atherosclerosis [8, 14].

The use of natural substances with antioxidant and hypoglycemic properties has been studied extensively. Studies have shown that antioxidant supplementation is able to alleviate chronic degenerative diseases such as diabetes mellitus and its associated complications [15–17].

Antioxidants are stable substances that act as reducing agents capable of reducing oxidized substances and making them stable, thus repairing the structure of the cellular macromolecules [18, 19].

N-Acetylcysteine (NAC) has antioxidant characteristics because its structure includes a sulfhydryl radical, which is responsible for the direct scavenging of oxidizing agents. It can also act indirectly by providing cysteine for glutathione synthesis, which is involved in the neutralization of reactive oxygen species [20–22].

Studies have shown that NAC is capable of lowering blood glucose levels, probably due to its ability to induce the production and secretion of insulin [23, 24]. Furthermore, there are reports that NAC reduces the severity of atherosclerosis by stabilizing the development of atherosclerotic plaque [22]. Diniz et al. [25] and Novelli et al. [26] showed that administration of NAC decreased the concentration of cholesterol and its lipoprotein fractions, and triacylglycerols. Meanwhile, the authors reported that HDL-cholesterol levels increased in the presence of a high calorie diet.

Therefore, it was hypothesized that NAC improves nutritional parameters and attenuates dyslipidemia and glycemia in the diabetic state.

The purpose of this study was to evaluate the beneficial effects of NAC on the lipid profile and atherogenic index in rats with experimentally induced T1DM.

## 2. Material and Methods

### 2.1. Animals and Experimental Procedures

This study was conducted according to the protocol approved (number 706) by the Ethics Committee on the Use of Animals (CEUA) at the Institute of Biosciences of Botucatu, University of São Paulo State (UNESP). 32 male Wistar rats aged 60 days with ±250 g body weight were housed in polypropylene cages in an acclimatized room (relative humidity 55 ± 5%; 23 ± 2°C and a dark-light cycle of 12 h). All animals received commercial feed (Purina, Campinas, Brazil; contained 22.0% protein, 3,8% fat, 44.5% carbohydrate, and 3.0 kcal/g of metabolizable energy) and water* ad libitum* during the experimental period of 37 days. Water and food intake was controlled daily between 9:00 and 10:00 a.m.

The animals were randomly divided into four experimental groups (*n* = 8): CTRL: control rats; CTRL+NAC: control rats treated with N-acetylcysteine; DM: diabetics rats; DM+NAC: diabetics rats treated with N-acetylcysteine.

In groups DM and DM+NAC, T1DM was experimentally induced by the administration of streptozotocin (STZ; 60 mg kg^−1^ b.w in single dose, ip.), diluted in 0.1 M sodium citrate buffer, pH 4.5. After 48 hours, the animals with a blood glucose concentration ≥ 250 mg dL^−1^ were considered diabetic according to Santos et al. [27]. CTRL+NAC and DM+NAC animals received NAC (25 mg kg^−1^ b.w. day^−1^) via gavage, for 37 days [28].

### 2.2. Nutritional Parameters

Nutritional parameters were estimated using the formulae according Kawahara et al. [29]: energy intake = mean food consumption × dietary metabolizable energy (3.8 kcal/g); alimentary efficiency = weight gain/energy intake; carbohydrate consumption = mean daily consumption of food × percentage of food carbohydrate; protein consumption = mean daily consumption of food × percentage of food protein.

### 2.3. Preparation of Serum and Cardiac Samples

At the end of the experimental period, the animals were fasted overnight for 12 h, anaesthetized with mixture (v/v) of ketamine chloride (10%)/xylazine chloride (2%) and euthanized by cervical decapitation. Blood Serum was obtained by centrifugation (1,400*g*/10 min) to determine the glycemia and lipid profile. Two samples of 100 mg each from the left ventricle of cardiac tissue were separated for biochemistry determinations.

### 2.4. Analytical Procedure

The concentration of glucose was determined by the enzymatic-colorimetric method, after incubation (37°C) with glucose oxidase/peroxidase and obtaining the final product quinoneimine [30].

Serum triacylglycerol was assayed by previous hydrolysis with the release of free fatty acids and glycerol, which under the action of glycerol kinase and peroxidase becomes in quinoneimine, which is directly proportional to triacylglycerol levels in the sample [31].

Total cholesterol levels were measured in the presence of the cholesterol esterase/oxidase according Moura [32]. VLDL-cholesterol (very low-density lipoprotein cholesterol) was obtained using the Friedewald formula [33]. HDL-Cholesterol (high-density lipoprotein cholesterol) was measured after selective precipitation of the serum lipoproteins (LDL-cholesterol and VLDL-cholesterol) using phosphotungstic acid and dispersed by centrifugation [34].

Dehydrogenase lactate activity was determined using the enzymatic kinetic method, according to oxidation rates of NADH [35].

The atherogenic index was calculated as demonstrated by Takasaki [36], AI = (cholesterol total − HDL-cholesterol)/HDL-cholesterol.

Samples of the left ventricle were homogenized in the presence of a sodium phosphate buffer (0.1 M, pH 7.4) and centrifuged (10,000 ×g/15 min). The supernatant determined the phosphofructokinase (PFK) activity in media containing glyceraldehyde 3-phosphate dehydrogenase, aldolase, triose phosphate dehydrogenase, NADH, ATP, and fructose 6-phosphate [37].

The total protein concentration was determined using the colorimetric method in the presence of a biuret reagent with color product released, the intensity of which was measured using a spectrophotometer [32].

To determine the myocardial glycogen, a sample of approximately 100 mg was homogenized in perchloric acid and the supernatant was submitted to be treated with amyloglucosidase according to the method used by Roehrig and Allred [38].

### 2.5. Statistical Analysis

All the data were statistically analyzed using a one-way ANOVA and Tukey's test to compare the means of the groups. A probability of 0.05 or less indicated that it was significantly different [39].

## 3. Results

Table 1 shows that glycemia, energy intake, carbohydrate, and protein consumption were increased (*p* < 0.05) in DM, when compared with the others. Alimentary efficiency in untreated diabetic rats (DM) was lower (*p* < 0.05) than DM+NAC. Control rats (CTRL and CTRL+NAC) showed higher values than diabetic rats (DM and DM+NAC).

In Table 2, it is possible to observe that the concentration of total proteins and glycogen in diabetic animals (DM) decreased (*p* < 0.05), and treatment with NAC improved (*p* < 0.05) these parameters. PFK activity in myocardium of diabetic rats (DM) was recovered (*p* < 0.05) by NAC administration (DM+NAC), reaching the values of CTRL group. There was no statistical difference in serum lactate dehydrogenase activity between the groups (*p* > 0.05).

Table 3 shows that NAC administration decreased serum concentration of triacylglycerols, total cholesterol, VLDL-cholesterol, and LDL-cholesterol (*p* < 0.05) and increased HDL-cholesterol concentration in diabetic rats (DM+NAC) compared to the DM group (*p* < 0.05). The DM+NAC group had a lower (*p* < 0.05) atherogenic index than the DM group.

## 4. Discussion

Streptozotocin administration is a classic model for experimentally inducing T1DM, because it selectively destroys *β*-pancreatic cells [40, 41]. It reaches the intracellular environment by glucose transporters and promotes the alkalization and subsequent fragmentation of DNA, which is repaired by poly-ADP-ribose polymerase, thus reducing NAD^+^ levels and depleting cellular ATP, causing cell death [42].

However, insulin release is reduced (not shown) and consequently the establishment of hyperglycemia, confirming decreased insulin serum concentration and elevated serum glucose in diabetic animals in this study (DM) (Table 1). These results were corroborated by Fernandes et al. [43].

The increased energy intake in diabetic rats (DM) (Table 1) is related to the increased consumption of carbohydrates and protein, indicating higher feed consumption. Although more feed was consumed, there was no increase in body weight, implying a decrease in feed efficiency (Table 1), which suggests a lower utilization of oxidizable substrates and could contribute to the hyperglycemia observed in the diabetic animals [44].

NAC administration in diabetic rats (DM+NAC) was shown raise insulin (not shown) and reduced hyperglycemia (Table 1). Ammon et al. [45] and Roma et al. [46] showed that administration of NAC in vitro improved insulin production and secretion in pancreatic *β* cells. Hyperglycemia decreased in treated diabetic rats (DM+NAC) (Table 1) and consequently improved the utilization of nutritional intake with a reduced energy intake and increased feed efficiency, accompanied by a reduced consumption of carbohydrates and proteins (Table 1).

In this study, it was possible to observe a cardiac reduction in the concentration of total proteins and glycogen in animals from group DM (Table 2), due to the severe catabolic state caused by T1DM. These results corroborate with the existing literature, which describes a special metabolism in the diabetic state where glucose is not used as an oxidizable substrate, thus activating the biochemical process gluconeogenesis, which is supported by a supply of *α*-keto acids [7, 47, 48].

The PFK catalyzes the irreversible conversion of fructose 6-phosphate into fructose-1-6-bisphosphate during glycolysis, making it a key enzyme for evaluating the speed of this metabolic pathway [49].

PFK activity was lower in group DM (Table 2), indicating reduced glycolysis in the diabetic state, even with less glycogen in these animals, probably due to the reduced synthesis of this polysaccharide by the cardiac tissue [50, 51].

Lactate dehydrogenase (LDH), a widely distributed enzyme found in the cytosol, catalyzes the reduction of pyruvate to lactate and is of fundamental importance in processes involving glycidic metabolism in anaerobiosis. Elevated serum levels in the plasma are important indicators of heart lesions, which cause serums to leak into the bloodstream [47, 52].

In this study, LDH serum activity showed no change (Table 2), indicating that a diabetic state does not damage heart tissue enough to increase the activity of this enzyme in the extracellular environment. However, other studies have identified increased LDH activity in blood plasma under diabetic conditions [53].

The administration of NAC promoted an increased concentration of proteins and glycogen in diabetic animals (Table 2), probably due to greater cellular utilization and internalization of glucose, since insulin release is higher in the presence of NAC [45, 46].

The improved glucose oxidation in diabetic and nondiabetic animals treated with NAC is proven by the increased PFK in the heart tissue (Table 2).

In this paper, there was an increase in total cholesterol, LDL-cholesterol, triacylglycerols, VLDL-cholesterol, and low HDL-cholesterol in diabetic rats (DM) (Table 3), confirming the development of dyslipidemia in this pathology, which is supported by previous studies [54, 55].

T1DM causes an insulin deficiency, resulting in increased lipolysis and subsequent *β*-oxidation of acetyl-CoA, which can be used in lipogenesis together with HMG-CoA reductase, a key enzyme in cholesterol biosynthesis [55], promoting the hepatic formation of VLDL-cholesterol and consequently increasing serum levels of cholesterol and LDL-cholesterol. Fatty acids that are not *β*-oxidated are esterified into triacylglycerols, which are incorporated into VLDL-cholesterol in the liver and exported to the bloodstream [10].

These metabolic events increase lipoproteins to a value above normal, identifying dyslipidemia as a risk factor for the development of atherosclerosis [55].

There is a correlation between dyslipidemia and hyperglycemia because LDL-cholesterol is easily glycated and is more susceptible to oxidation in the subendothelial layer [56].

Glycated LDL-cholesterol crosses the endothelial layer and interacts with oxidizing agents, turning it into oxidized LDL, identified as an invasive agent that promotes the recruitment of macrophages, resulting in foam cells and the consequent formation of atherosclerotic plaque [8, 14].

In addition, the regulatory processes of hyperglycemia produce oxidizing agents and inflammatory mediators that are contributing factors in the formation of atherosclerotic plaque [12, 13].

Considering the (total-cholesterol − HDL-cholesterol) : HDL-cholesterol ratio, the atherogenic index was elevated in diabetic rats (DM) (Table 3) due to the dyslipidemia that is characteristic of this pathology.

Administration of NAC decreased serum levels of triacylglycerols, VLDL-cholesterol and LDL-cholesterol in diabetic rats (DM+NAC) (Table 3), while increasing the concentration of HDL-cholesterol.

Considering that cell recognition between apolipoproteins and membrane receptors depends on their structure being intact, the antioxidant action of NAC can prevent oxidation of proteins and ensure endocytosis of lipoproteins, decreasing its serum concentration [57].

The improved dyslipidemia in diabetic rats treated with NAC (DM+NAC) (Table 3) can be explained by the greater insulin secretion in the presence of this antioxidant, since insulin decreases blood sugar levels and lipolysis in adipose tissue. Studies have shown that NAC increases insulin secretion [45, 46]. On the other hand, the presence of insulin increases the activity of lipoprotein lipase, which catalyzes the breaking of triacylglycerol ester bonds, increasing the clearance of VLDL-cholesterol [55, 58].

In this paper, HDL-cholesterol levels increased in diabetic rats treated with NAC (DM+NAC) (Table 3). These results are corroborated by Almeida et al. [54]. In addition, the increased insulin level elevates the activity of lecithin-cholesterol acyltransferase (LCAT) and the enzyme responsible for extracellular cholesterol esterification, thus increasing the efficiency of reverse cholesterol transport, indicating an inverse correlation with cardiovascular accidents [8, 55].

Studies have identified a relationship between antioxidants and reduced cholesterol levels, due to inhibition of HMG-CoA reductase activity and cholesterol biosynthesis [44, 55].

Thus, the NAC in diabetic rats was able to normalize the atherogenic index because it was possible to control the lipid profile, a finding that is corroborated by Yang et al. [58], who noted that an increased atherogenic index is related to low antioxidant activity.

Thus, NAC can help to reduce the formation of atherosclerotic plaque by lowering blood glucose levels, the glycation of LDL-cholesterol, and its consequent oxidation. Additionally, Sung et al. [59] reported that NAC is capable of reducing the expression of gene CD36, the gene involved in the formation of foam cells, another factor that can help to reduce the development of atherosclerotic plaque.

## 5. Conclusion

NAC treatment improves the glycemia and nutritional parameters induced by T1DM, and this antidiabetogenic agent contributes to reducing diabetic complications such as dyslipidemia and atherosclerosis by improving the atherogenic index.

## Figures and Tables

**Table 1:** Glycemia, energy intake, alimentary efficiency, carbohydrate, and protein consumption in diabetic rats that have been treated and not treated with NAC.

Parameters	Groups			
	CTRL	CTRL+NAC	DM	DM+NAC
Glycemia mg/mL	92.71 ± 14.41^a^	98.10 ± 14.07^a^	351.04 ± 27.94^c^	128.84 ± 12.87^b^
Energy intake Kcal/day	98.33 ± 1.50^a^	98.88 ± 2.02^a^	160.66 ± 9.27^c^	143.82 ± 19.12^b^
Alimentary efficiency g/Kcal	1.44 ± 0.11^c^	1.56 ± 0.12^c^	0.33 ± 0.21^a^	0.65 ± 0.17^b^
Carbohydrate consumption g/day	22.09 ± 0.34^a^	22.21 ± 0.45^a^	36.09 ± 2.08^c^	32.31 ± 4.30^b^
Protein consumption g/day	0.29 ± 0.01^a^	0.30 ± 0.01^a^	0.48 ± 0.33^c^	0.43 ± 0.06^b^

Data is mean ± SD (*n* = 8). ^a, b, c^In each row, the mean value followed by different letter indicates a statistically significant difference (*p* < 0.05). Control rats (CTRL); control rats treated with N-acetylcysteine (CTRL+NAC); diabetic rats (DM); diabetic rats treated with N-acetylcysteine (DM+NAC).

**Table 2:** Biochemical characteristics in diabetic rats that have been treated and not treated with NAC.

Parameters	Groups			
	CTRL	CTRL+NAC	DM	DM+NAC
Total protein mg/100 mg tissue	33.70 ± 1.40^c^	30.47 ± 0.98^b^	25.37 ± 1.08^a^	31.01 ± 1.43^b^
Glycogen mg/g tissue	34.50 ± 3.40^c^	33.04 ± 3.60^c^	16.16 ± 2.58^a^	27.01 ± 3.76^b^
Phosphofructokinase *η*mol/g tissue	1.67 ± 0.18^b^	2.27 ± 0.71^c^	0.69 ± 0.10^a^	1.45 ± 0.30^b^
Lactate dehydrogenase U/L	67.29 ± 10.59	67.80 ± 9.86	66.78 ± 11.65	59.70 ± 6.40

Data is mean ± SD (*n* = 8). ^a, b, c^In each row, the mean value followed by different letter indicates a statistically significant difference (*p* < 0.05). Control rats (CTRL); control rats treated with N-acetylcysteine (CTRL+NAC); diabetic rats (DM); diabetic rats treated with N-acetylcysteine (DM+NAC).

**Table 3:** Lipid profile in diabetic rats that have been treated and not treated with NAC.

Parameters	Groups			
	CTRL	CTRL+NAC	DM	DM+NAC
Triacylglycerol mg/L	153.46 ± 25.47^a^	161.30 ± 25.72^a^	312.70 ± 33.67^b^	171.30 ± 26.79^a^
Total cholesterol mg/L	98.24 ± 10.84^a^	98.62 ± 15.27^a^	150.38 ± 18.64^b^	109.33 ± 10.01^a^
VLDL-cholesterol mg/L	30.69 ± 5.09^a^	32.26 ± 5.14^a^	62.54 ± 6.73^b^	34.26 ± 5.36^a^
LDL-cholesterol mg/L	29.07 ± 8.17^a^	30.13 ± 10.43^a^	68.66 ± 16.14^b^	30.52 ± 10.45^a^
HDL-cholesterol mg/L	38.48 ± 6.59^b^	36.23 ± 8.49^b^	19.18 ± 4.28^a^	44.54 ± 7.82^b^
Atherogenic index	1.60 ± 0.47^a^	1.66 ± 0.30^a^	7.11 ± 1.63^b^	1.39 ± 0.19^a^

Data is mean ± SD (*n* = 8). ^a, b, c^In each row, the mean value followed by different letter indicates a statistically significant difference (*p* < 0.05). Control rats (CTRL); control rats treated with N-acetylcysteine (CTRL+NAC); diabetic rats (DM); diabetic rats treated with N-acetylcysteine (DM+NAC).
